# Personal protective equipment: Shortage or waste?

**DOI:** 10.1017/ice.2020.354

**Published:** 2020-07-22

**Authors:** Dayana Souza Fram, Daniela Vieira da Silva Escudero, Luciana de Oliveira Matias, Wanderson Eduardo Gomes de Souza Coelho, Thaysa Sobral Antonelli, Diogo Boldim Ferreira, Eduardo Alexandrino Medeiros

**Affiliations:** Division of Infection Control and Hospital Epidemiology, Hospital São Paulo, Universidade Federal de São Paulo, São Paulo, Brazil

*To the Editor*—With the progression of the coronavirus disease 2019 (COVID-19) pandemic, the personal protective equipment (PPE) shortage has been highlighted.^1^ The sudden increase in demand for PPE due to the number of COVID-19 cases, misinformation, panic buying, and stockpiling resulted in global shortages. The World Health Organization (WHO) published a guideline for the rational use of PPE for coronavirus disease in healthcare and home-care settings during severe shortages.^2^ Despite the importance of this topic, observational studies that evaluate the use of PPE during the pandemic by healthcare workers (HCWs) are scarce. A Chinese cross-sectional survey using a self-administered questionnaire included 1,357 HCWs and showed that 89% had sufficient knowledge and 89.7% followed correct practices concerning severe acute respiratory coronavirus virus 2 (SARS-CoV-2).^3^

Researchers in the division of infection control and hospital epidemiology of a teaching hospital in Brazil observed compliance regarding additional transmission-based precautions in exclusive care units for patients suspect or confirmed to COVID-19 from April 1 to May 15, 2020. The institutional protocol to control the coronavirus disease was developed based on guidelines of the World Health Organization (WHO) and the National Health Surveillance Agency (ANVISA), a regulatory body of the Brazilian government.^4,5^ Prior to the onset of observations, healthcare professionals (HCPs) received face-to-face or video training on SARS-CoV-2 precautions. In assistance activities, HCPs and support teams should follow contact and droplet precautions or contact and airborne precautions for aerosol-generating procedures.

Compliance was considered as satisfactory when the HCPs wore all 5 proper PPE (ie, gown, eye protection, head cap, mask, and gloves) recommended for each specific procedure. During the study, 260 observations were performed and the compliance rate was 31.5% (n = 82). The compliance rate was 22% (2 of 9) among physiotherapists, 29% (15 of 52) among physicians, 31% (56 of 182) in the nursing team, and 53% (9 of 17) among all others (ie, nutrition team, occupational therapists, X-ray technicians, and cleaning staff). More than 1 improper PPE use was identified in each observation, totaling 322 failures, of which 40% (n=129) were practices that could have resulted in self- and/or environmental contamination. Furthermore, 60% of these failures (n=193) were practices that resulted in waste of PPE (Table 1).

**Table 1.** Distribution of Noncompliance to PPE Use in Exclusive Care Units for Patients Suspect or Confirmed to COVID-19 in a Teaching Hospital in Brazil, 2020

Variable	No.	%
**Practices that could have resulted in self and/or environmental contamination**	129/322	40
Use of potentially contaminated PPE outside the assistance area	44	34.1
Professional did not use available PPE	63	48.9
Overuse of PPE with risks of exposure to microorganisms^a^	7	5.4
Inappropriate PPE with risks of exposure to microorganisms^b^	12	9.3
Inadequate handling of PPE^c^	3	2.3
**Practices that resulted in waste of PPE**	193/322	60
Overuse of PPE	84	43.5
Inappropriate and more expensive PPE than indicated^d^	109	56.5
**Total**	322	

Note. PPE, personal protective equipment.

a Surgical mask under FFP2/N95.

b Gowns had less protection than recommended.

c Adjusted the mask in the assistance area.

d An N95/FFP2 mask instead of a surgical mask; gowns had higher protection than recommended.

These preliminary results suggest unnecessary consumption of PPE by HCPs, contributing the shortage of these products, which may put the safety of professionals and patients at risk. Therefore, determining the cause of this behavior is crucial to developing targeted interventions to increase precaution compliance to control the spread of SARS-CoV-2.
